# Relation between increasing attachment security and weight gain: a clinical study of adolescents and their parents at an outpatient ward

**DOI:** 10.1007/s40519-023-01611-x

**Published:** 2023-10-10

**Authors:** Christina M. E. Gezelius, Björn A. Wahlund, Britt M. Wiberg

**Affiliations:** 1 Department of Children and Youth Psychiatry, Center for Clinical Research, Falun Hospital, Falun, Sweden; 2 Department of Clinical Neuroscience, Karolinska Institute, Stockholm, Sweden; 3 California Institute of Technology, Pasadena, USA; 4 Department of Psychology, Umeå University, 90187 Umeå, Sweden

**Keywords:** Attachment, Eating disorders, Adolescents, Parents, Clinical study

## Abstract

**Purpose:**

We wanted to evaluate the impact of a relational focus in the treatment of adolescent ED-patients and their parents at an intensive outpatient ward, based on attachment theory, combined with a family approach and psychodynamic principles. Our aim was to investigate the distribution of different attachment styles among the adolescent ED-patients and their parents, and to find out if they could change by the treatment.

**Methods:**

Swedish adolescents (*n* = 33; 3 boys, 30 girls) and their parents (*n* = 60; 34 mothers, 26 fathers) participated. Measures: Attachment Style Questionnaire (ASQ), Body Mass Index (BMI) and Children’s Global Assessment Scale (CGAS) before and after treatment.

**Results:**

The adolescents were high on Need for Approval (ASQ4) of the Insecure/Anxious scale before treatment (in contrast to the parents). The patients had a significant decrease in ASQ4 after treatment, which correlated inversely to the increase in BMI but not to CGAS. The mothers showed features of the Secure/Confident style, fathers of the Insecure/Avoidant with elevated Relationships as Secondary (ASQ2).

**Conclusions:**

Treatment with a relational and a family focus has impact on attachment insecurity in adolescent ED-patients and outcomes in terms of BMI. It is important to engage the parents, who need to help the adolescents to separate at that developmental stage. A secure therapeutic context, which enables mentalizations and allows new relational experiences, is essential. The ASQ-instrument is useful in indicating how the treatment of ED-adolescents is proceeding.

**Level of evidence:**

Level IV: evidence obtained from multiple time series with the intervention.

## Introduction

Eating Disorders (EDs) constitute a great health problem. They are in many ways disabling and common—especially in females—and in increasing numbers [1]. Anorexia Nervosa (AN) has the highest death rate of all psychiatric disorders [2] and a poor longtime prognosis for full symptom recovery [3]. Nevertheless, articles on EDs are published less frequently [4] and in less high-ranked journals compared to other mental illnesses, exposing the need for more research on these disturbances. Clinical studies before and after treatment also including parents are scarce.

The origin and the maintaining factors of an ED are complex. Although handling skills and cognitive aspects are important parts of therapy, they are not enough [5, 6]. The onset of EDs takes place mainly during adolescence [1], when great physical and challenging psychological steps need to be taken towards adulthood, suggesting the importance of developmental aspects. Early experiences of supporting relationships or not and adverse stressful life events influence the process to young adulthood [7].

Attachment theory formed by John Bowlby and Mary Ainsworth [8, 9], states that there is a connection between early experiences of caregiving and personality in later life. They are the basis of *Internal Working Models*, which influence psychological functioning and can be categorized in different styles. The distribution of the five-factor structure of the ASQ decides the person’s interpersonal style [10]. ASQ measures five factors: *Discomfort with Closeness (ASQ1)* and *Relationships as Secondary (ASQ2)* assess the “*Insecure/Avoidant*” scale; *Confidence (ASQ3)* the “*Secure/Confident*” scale; *Need for Approval (ASQ4)* and *Preoccupation with Relationships (ASQ5)* the “*Insecure/Anxious/Ambivalent*” scale, all three styles being organized. A fourth style *Insecure/Fearful* is characterized by being disorganized and showing traits of the other styles depending on the situation [11]. It is connected to experiences of fear and loss.

Secure attachment pattern relates to the abilities in identifying and describing emotions and in regulating them [12, 13]; so-called *mentalization*. It is an innate human capacity. It takes considerable environmental input for it to develop fully and in a balanced way. Mentalizing is needed not only by parents in the attachment context, but also by family and the broader social environment [14].

When the emotional arousal is of too high intensity and/or of long duration, relative to the individual’s capacity to mentalize, attempts to influence the experience and expression of emotion are not sufficient. The ability to understand the intentions behind actions from themselves and others breaks down and leaves sensitive individuals vulnerable to communicate through action instead of words, including somatization. According to Haynos and Fruzzetti [15] AN may be described as a disorder of emotional dysregulation.

There is much evidence now that ED-patients are mainly insecurely attached [16, 17] and that they have difficulties in emotion regulation and mentalization. Body dissatisfaction is most consistently associated with an increase in Need for Approval (ASQ4)—an aspect of anxious attachment [18, 19] in the Attachment Style Questionnaire (ASQ) [10]. Dysfunctional parental attachment was related to lower body satisfaction in adolescents in a study by Szalai et al. [20]. Illing et al. [21] found that higher anxious attachment pre-treatment was significantly related to greater ED-symptom severity and poorer treatment outcome in all different EDs. Amianto et al. [22] concluded that attachment strength may contribute to protection from developing an ED, when found that healthy siblings of women with AN were securely attached measured by ASQ. They reported simultaneously lower maternal care and overprotection like their affected sibling. Gale et al. [23] made a review on the father–child relationship in the development and maintenance of adolescent AN and Bulimia Nervosa (BN): fathers play a significant role in fostering a child’s sense of autonomy. A large prospective study of adolescents from preadolescence showed, that better attachment to the mother led to less pronounced disturbed eating [24].

In a previous study by Gezelius et al. [25] all ED-adolescent patients had negative self-image measured by Structural Analysis of Social behavior (SASB) [26, 27] before treatment at an intensive outpatient ward and changed to positive after. They also had a significant increase in weight measured by Body Mass Index (BMI) [28] and in Children’s Global Assessment Scale (CGAS) [29]. The parents’ self-image was mainly positive from the beginning and did not change. From the same clinical sample, attachment data measured by ASQ were collected from both adolescent ED-patients and their parents before and after treatment.

## Aims

The main aim of this clinical study was to explore the attachment styles, using the ASQ-questionnaire, of adolescent patients with ED diagnosis and their parents before and after treatment at an intensive outpatient ward with a family and relational focus. Another aim was to relate the attachment styles of the patients to the outcome measures of BMI and CGAS.

## Methods

### The setting of the study and context of the treatment

This intensive outpatient program had a duration of 16 ± 2 months. In the beginning, the treatment was concentrated on connecting with the adolescent ED-patient and her/his parents and to establish good regular eating habits at the day-care unit and to handle anxiety to attain full nutrition. Every patient together with their respective parents had their own mini team: one therapist (a social worker or a psychologist) and two staff members. One of the latter was sitting beside the patient as a support during meals but also helping to put words to feelings and thoughts. Initially, the patient spent every weekday at the unit, then in diminishing frequency until being mere an outpatient. Guidance was given to the parents at meetings with the whole family every week to apply the same practice at home. It sometimes happened, that the patient needed tube feeding and hospitalization because of a total refusal to eat or because of a very bad somatic condition. The team kept in contact with the patient, collaborated with the hospital staff, and facilitated a step-by-step return to the daycare unit. The somatic status of the patients often causes much worry. For the staff, including the medical part, to feel safe, a backup from the outside was arranged. A pediatrician, always the same person, attended the intake meeting and came to meetings weekly, made medical checks initially and when needed. A dietician was also a regular consultant. When good eating routines were established, more effort was put into relational and emotional matters, both in the family, within the individual, and to others. Regular family therapy was given based on psychodynamic principles and attachment theory together with cognitive elements. No manuals were used. The family sessions were held by one therapist and one staff member, who was close to the patient’s daily life on the ward, and together they established a reflective functioning atmosphere and dialogue.

Every month joint supervision was given a whole day to all staff members, including therapists, social workers, nurses, psychiatrist, and psychologist. All patients stayed at home that day. A holding environment to the staff was created. Attachment theory and psychodynamic principles constituted the base and were then applied on the cases, guided by the initial self-reports of the family. A continual learning process was established.

### Study design

The study has an observational cohort design and “research-quality naturalistic data” [30] were used, based on a clinical sample of adolescent ED-patients and their parents without a control group. The patients received treatment according to the above account and were included in the research study between May 2004 and May 2010, with just a few patients in the beginning when the unit was set up.

### Participants

All adolescent patients and their mothers and fathers were asked to answer the self-report questionnaires both at the start and end of the treatment, as part of a quality assurance database study. Later we decided to turn it into a research study. All participants were asked for consent. Some patients and parents did not want to be part of the research study and were marked as fall-offs together with a few with incomplete answers. The distribution of diagnoses among them was nearly the same as in the research group.

The final research sample included 33 patients (3 boys and 30 girls) between 12 and 17 years, mean age of 15.6 ± 0.7 years (mean ± standard error of the mean [SEM]) at admission. They were all inborn Swedes and economically relatively well-situated. The distribution of diagnoses using DSM-IV-TR [31] was: AN (*n* = 19; 58%), Eating Disorders Not Otherwise Specified of anorectic type (EDNOS-AN) (*n* = 14; 42%). All 60 parents participating in the final sample were biological, 34 mothers and 26 fathers, of which 28 mothers and 24 fathers had participating adolescents. The majority lived together. Eight mothers and four fathers lived separately from the other parent. Three patients did not have a participating parent in the study, eight had either of the two, and 22 had both.

## Instruments

*Attachment Style Questionnaire* (ASQ) [10] is a self-report instrument for measuring attachment among adolescents and adults based on attachment theory [8, 9]. The questionnaire consists of 40 items with a 6-point scale, ranging from 1 (totally disagree) to 6 (totally agree).

The ASQ has high validity and reliability along with good concurrent validity with other measures of adult attachment according to Hazan and Shaver [32] and confirmed by Levy and Kelly [33]. The Swedish version [34] is translated and tested on samples of students (*n* = 90) and of patients from both somatic and psychiatric clinics (*n* = 66). The scales correlated similar to each other compared to the original version. The Cronbach’s alpha for the five scales ranged from 0.71 to 0.84. Validity tests by Tengström and Håkanson [35] replicated the three attachment patterns: secure, insecure/avoidant, and insecure/anxious, like the original version of ASQ.

*Body Mass Index* (BMI) [28] is defined as the person’s weight in kilograms, divided by the square of the person’s height in meters. Using the cutoff points of the World Health Organization normal BMI-values increase with the child’s age.

*Children’s Global Assessment Scale* (CGAS) [29, 36] is a tool based on clinical assessment of the global functioning of patients aged 4–20 years. The scale is continuous, ranging from 0 to 100, where 100 stands for very good functioning in every aspect. The raters note the most impaired level of general functioning at a specified period. Concurrent validity is shown by Bird et al. [36]. Best reliability and accuracy are obtained by experts and “moderate” with untrained raters [37, 38].

An instrument assessing eating psychopathology would have been appropriate in this study and was also used at the unit. However, when becoming a member of the internet-based collection system for specialized ED-treatment units in Sweden, we had to change instruments during the ongoing project.

### Procedure

On the first day of treatment, the ASQ-questionnaires were administered to both the adolescent patients and their parents. The physical examination of the patients, including weight, was performed after the questionnaires were completed and the BMI was calculated. At the second last meeting before the end of the treatment, the same procedure took place. CGAS was performed within 2 weeks of getting to know the patients and again at the end of the treatment. A child and youth psychiatrist diagnosed the patients using DSM-IV-TR [31].

### Statistical analysis

All statistical and mathematical calculations were performed using the program by JMP® version 10.0.2. On each sample of the family members, we performed calculations of mean ± Standard Error of Mean (SEM) of the ASQ1–5. All variables fitted normal distributions in each group of family members before and after treatment. Analysis of Variance and Student tests were used to test the difference between means. The significant levels in the statistical analyses were set to *p* < 0.05, *p* < 0.01, and *p* < 0.001.

In our earlier published study [25] on the same ED-patients, we presented demographic data, BMI and CGAS before and after treatment. The mean values ± SEM of BMI were 17.5 ± 0.4, *n* = 33 and 19.5 ± 0.4 (*p* < 0.05) *n* = 31 before and after and of CGAS 51.1 ± 2.2, *n* = 32, and 69.7 ± 2.3 (*p* < 0.05) *n* = 31.

Furthermore, we studied relationships between differences in ASQ, ΔASQ, versus differences in BMI, ΔBMI, and CGAS, ΔCGAS, before and after treatment. Cross correlations (parametric) were performed on ΔASQ, ΔBMI, and ΔCGAS. We analyzed the correlation matrix and then examined linear regression models between ΔBMI and ΔCGAS (*y*-variables) and ΔASQ-variables (*x*-variables; one by one *x*-variable, while the ASQ1–5 were correlated). By the distribution of the points in the plot between ΔASQ4 and ΔBMI we constructed a four-field table, i.e., a cross-tabulation. We used the axes to build four quadrants with origo in zero and identified both variables increase and decrease. Then, we could easily test differences between the numbers of individuals in each quadrant by Chi-square analysis.

## Results

### Means of ASQ1–5 of the family members

The means of ASQ1–5 of adolescent patients and their mothers and fathers are shown in Table 1. The mothers displayed features of the Secure/Confident scale, fathers of the Insecure/Avoidant with elevated ASQ2—found by dimensional assessment of the ASQ [34]. The adolescents had high scores on ASQ4 of the Insecure/Anxious scale.

**Table 1:** Mean and standard error of mean (SEM) of Attachment Style Questionnaire (ASQ) in adolescents and parents before and after treatment

Family members	Treatment	ASQ1	ASQ2	ASQ3	ASQ4	ASQ5
Adolescents						
*n* = 33	Before	3.54 ± 0.17	2.86 ± 0.13	4.05 ± 0.17	4.04 ± 0.20**	3.52 ± 0.17
*n* = 30	After	3.12 ± 0.20	2.32 ± 0.15	4.41 ± 0.19	3.35 ± 0.18**	3.30 ± 0.18
Mothers						
*n* = 34	Before	2.93 ± 0.15	2.21 ± 0.10	4.62 ± 0.11	3.03 ± 0.14	3.02 ± 0.13
*n* = 31	After	2.84 ± 0.15	2.15 ± 0.11	4.77 ± 0.12	2.91 ± 0.14	2.86 ± 0.16
Fathers						
*n* = 26	Before	3.12 ± 0.14	2.70 ± 0.15	4.39 ± 0.12	2.86 ± 0.15	3.06 ± 0.14
*n* = 21	After	3.10 ± 0.12	2.50 ± 0.16	4.48 ± 0.13	2.69 ± 0.15	2.95 ± 0.18
Ref. adolescents						
*n* = 74		3.1 ± 0.10	2.0 ± 0.07	4.4 ± 0.09	3.5 ± 0.10	3.5 ± 0.09
Ref. adults						
*n* = 90		3.23 ± 0.10	2.10 ± 0.06	4.42 ± 0.08	3.47 ± 0.10	3.52 ± 0.08

Reference groups from the validation of the Swedish version [34, 35]. One-way ANOVA

Test of mean before and after by Analysis of Variance (ANOVA)

ASQ1: discomfort with closeness; ASQ2: relationships as secondary; ASQ3: confidence; ASQ4: need for approval; ASQ5: preoccupation with relationships

**p < 0.01, significant decrease in ASQ4 in adolescents

### Means of ASQ1–5 of the adolescent ED-patients and parents after treatment

**Fig. 1:**
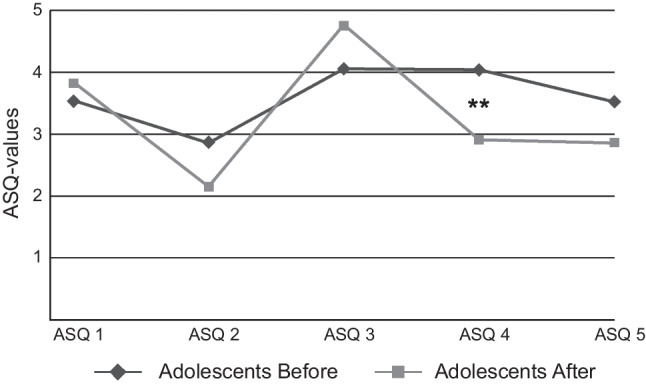
Attachment Style Questionnaire (ASQ)-scales in adolescent ED-patients before and after treatment. ASQ1: discomfort with closeness; ASQ2: relationships as secondary; ASQ3: confidence; ASQ4: need for approval; ASQ5: preoccupation with relationships. *x*-axis: ASQ 1–5 means, *y*-axis: ASQ-values. **p < 0.01

Adolescent ED-patients had a significant decrease (*p* < 0.01) in ASQ4. They moved towards Confidence/Secure scale (Table 1, Fig. 1). The ASQ-scores of the parents remained almost the same after treatment.

### Clinical ratings in relation to ASQ, before and after treatment of the adolescents

An important aim of this study was to find out if there were any relationships between attachment values of ASQ and outcome measures of BMI and CGAS. There were no correlations or linear relations between ASQ 1–5 as compared with BMI and CGAS. However, by measuring the change in ASQ4 and BMI, we found an inverse linear relationship between these two variables. Thus, a change in ASQ4 (ΔASQ4) was defined as independent variable, *x*, whereas the corresponding change in BMI (ΔBMI) was defined as a dependent variable, *y*, *R* square = 0.14, *F* = 4,6, *p* < 0,05, *n* = 30. This means that a single ASQ4-value does not tell the BMI. The difference between two measurements of ASQ4 can tell the difference in BMI.

By cross-tabulation, 22 individuals were found in the upper left quadrant, where ASQ4 decreased, and BMI increased. In the upper right there were five individuals (increased ASQ4 and increased BMI) and only three in the lower right quadrant (increased ASQ4 and decreased BMI) and none in the lower left (decreased ASQ4 and BMI). We performed Chi-square calculation on the quadrants with individuals, even though it is doubtful to include the three. Still, they did not contribute substantially to the Chi-square value (Chi-square = 15.1, *df* = 1, *p* < 0.001, *n* = 30). Thus, the main part (73%) of all individuals lowered their ASQ4 and increased their BMI. There were no relations between ΔASQ1–5 and ΔCGAS.

### Change of ED-diagnosis after therapy

At the end of the project when 32 patients participated, 24 patients (*n* = 24; 75%) had no longer an ED-diagnosis. There were 3 AN (9.4%), and 5 EDNOS-AN (15.6%).

## Discussion

**Fig. 2:**
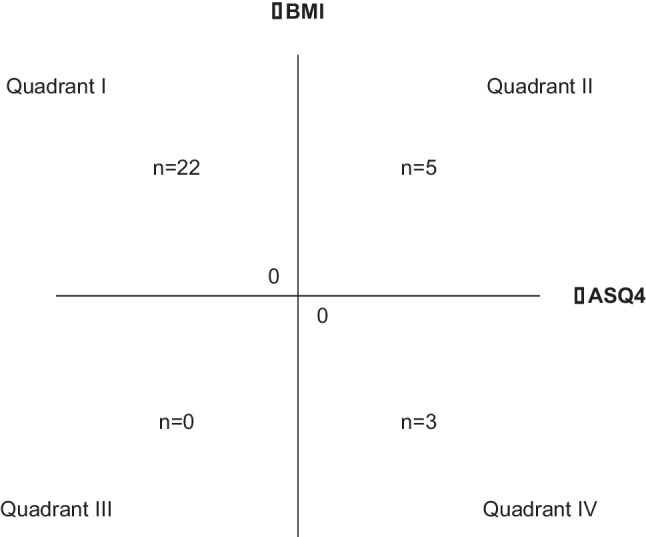
Cross-tabulation of ∆ASQ4 versus ∆BMI using zero points of the change of each variable. The cross denotes the two axes of ∆ASQ4 and ∆BMI, respectively. Origo is zero for both axes and denotes no change. The distribution is significant between the boxes (Chi-square = 15.1, *p* < 0.001)

The main aim of this clinical study was to explore the attachment profiles by the ASQ of adolescent patients with ED diagnosis and their parents before and after treatment with a family and relational focus at an intensive outpatient ward. After treatment the Adolescent ED-patients had a significant decrease (*p* < 0.01) in ASQ4 as shown in Table 1 and Fig. 2. They moved towards Secure/Confident style. Our hypothesis is that this could be an effect of the treatment’s relational focus, indicating new positive experiences.

Most of the mothers displayed a “supersecure” pattern with high ASQ3, and some among fathers and children. Adding up five items of the ASQ referring to self-valuation, and the sum exceeds the already high mean of the ASQ3 indicates according to Håkanson and Tengström overvaluation of the self [34], a narcissistic personality trait [39]. That implies less interest in and lower openness to others and a tendency to regard the child as a part of oneself, which might influence the mother–adolescent relationship.

Fathers in this study had elevated ASQ2 (Relationships as secondary, belonging to the Avoidant style), which may affect the marital as well as the father–child relationship. In our previous study on the same population [25], fathers scored low on self-protection according to SASB, which signifies problems with nurturing and protecting oneself as well as others.

With both parents distant, as our study proposes, a family climate is created, where the child is left alone without transforming moments of meeting [40], when the individual is seen just as is without preconceived ideas and fully acknowledged. The adolescent is left with a sense of not making an impact on others. A gap arises, which the adolescents try to bridge by being overly attentive to parents and others to get in contact. When this is not obtained, they anxiously increase their efforts, obsessively interpreting other’s minds, although not accurately, so-called *hypermentalizing* [41]. Disordered eating allows momentary relief in stressful times by acting towards oneself, but leading to a vicious/dysfunctional cycle, which interferes with the development of more adaptive emotion regulation strategies.

The father has an important role in helping a child during adolescence to separate from the mother and to individuate during normal psychological development, even more so if the mother wants to hold back. This task is more difficult to accomplish if the father is not sufficiently present in the life of the adolescent.

Another aim was to relate the ASQ-scales of the patients to the outcome measures of BMI and CGAS before and after treatment. In this study the rise of the BMI was directly and inversely related to the diminution of the ASQ4, implying a direct effect on the restrictive symptoms by establishing a new Internal Working Model [8], another way of relating to self and others. Insecure attachment, personality disorders, and most psychiatric disorders can be seen as manifestations of communicative strategies to ensure appropriate accommodation to changing situations [15]. Eating Disorders could be states of goal-directed behavior to regulate unbearable and unmentalized self-states, after successful treatment no longer needed. The increase in CGAS had no relation to the decrease in ASQ4 or any other rate on the ASQ-scale.

The foundation of the treatment at the ward was to be a holding environment for the patients, their parents, and the staff and to create self-consciousness in a mentalizing climate [7]. Validating emotions and putting them into words and connecting them to the cause, was a central part of therapy, during meals at the unit, in other daily situations and during therapy. The forms completed initially were valuable guiding tools. The staff engaged the patients by sharing meals and activities, served as experts as well as role models and initiated new ways of relating and reflecting to break the patients’ isolation. The family sessions were held by the family’s team. ED-focused family therapy is found to be the strongest evidence-based treatment for adolescent AN [42].

The ward became “a safe and mentalizing place” for the patients and even for the parents, who were seen as important actors in the recovery of their children. They took, with growing confidence, more and more responsibility in handling the hard situations during meals and in communicating with their adolescents. The patients got new relational experiences, ASQ4 was lowered, and they moved towards Secure/confidence attachment style. Their mentalizing capacity was growing, giving them the possibility to relate to themselves and to others in a new way. Starvation as an emotion regulation strategy was losing its importance allowing a break from the vicious cycle. Recovery of the patient’s and the family’s social functions became possible leading to salutogenic cycles [15]. The adolescent developmental crisis [43] got potential to be solved for future life by new experiences.

## Strengths and limits

Treatment with a relational and family focus impacts attachment insecurity in patients as well as outcome in terms of BMI among the adolescent ED-patients. The change in ASQ4 is significantly correlated with change in BMI. Attachment-styles of all the family members put light on the possible dynamics of interplay in families with adolescent ED-patients.

The main limitation concerns the rather small sample size, which also was the reason why the patients were not stratified into different groups according to gender or diagnosis. The difference in weight cutoff between anorexia and atypical anorexia is questioned by Monteleone and colleagues [44] when found that adolescents with atypical AN diagnosis do not differ from those with full AN diagnosis. They may even show greater psychopathology. The patients in our study received the same treatment irrespective of diagnoses. The fall-off was examined and found with small differences, both in distribution of diagnoses and patterns of attachment.

An instrument assessing eating psychopathology would have been appropriate to present in this study, but of reasons mentioned above it was not possible. No ED-diagnosis after therapy among a majority of the patients confirms the validity of our outcome measure BMI.

There was no control group, because this was a study in an authentic environment, but data were collected systemically. An observational cohort design and “research-quality naturalistic data” were used [30].

The long-time-standing result is not at hand, and a follow-up study is eligible. Further studies are necessary to explore more deeply the connection between attachment and self-image.

## What is already known on this subject?

Insecure attachment, mainly with Anxious and Fearful attachment styles, among ED-patients has been found [7, 45]. Body dissatisfaction is most consistently associated with an increase in ASQ4 [18, 19]—an aspect of Insecure/Anxious/Ambivalent attachment style. Greater ED-symptom severity and poorer treatment outcome is related to higher anxious attachment [21].

## What this study adds

Assessing the ASQ of the family members clarifies the family dynamics. ASQ4 is an important scale to estimate repeatedly in adolescent ED-patients to check if treatment progress is/is not obtained. The rise of the BMI was directly and inversely related to the diminution of the ASQ4, implying a direct effect on the restrictive symptoms by new ways of relating to self and others.

## Data Availability

The data sets generated and analyzed during the current study are available from the corresponding author on reasonable request.

## References

[1] Galmiche M, Déchelotte P, Lambert G, Tavolacci MP (2019). Prevalence of eating disorders over the 2000–2018 period: a systematic literature review. Am J Clin Nutr.

[2] Arcelus J, Mitchell AJ, Wales J, Nielsen S (2011). Mortality rates in patients with anorexia nervosa and other eating disorders: a meta-analysis of 36 studies. Arch Gen Psychiatry.

[3] Steinhausen HC (2002). The outcome of anorexia nervosa in the 20th century. Am J Psychiatry.

[4] Solmi F, Bould H, Lloyd EC, Lewis G (2021). On the limited visibility of eating disorders research. Lancet Psychiatry.

[5] Fairburn CG (2008). Cognitive behavior therapy and eating disorders.

[6] Strober M, Johnson C (2012). The need for complex ideas in anorexia nervosa: why biology, environment, and psyche all matter, why therapists make mistakes, and why clinical benchmarks are needed for managing weight correction. Int J Eat Dis.

[7] Tasca GA (2019). Attachment and eating disorders: a research update. Curr Opin Psychol.

[8] Bowlby J (1988). A secure base: parent–child attachment and healthy human development.

[9] Ainsworth MDS (1978). The Bowlby–Ainsworth attachment theory. Behav Brain Sci.

[10] Feeney JA, Noller P, Hanrahan M, Sperling MB, Berman WH (1994). Assessing adult attachment. Attachment in adults: clinical and developmental perspectives.

[11] Main M, Solomon J, Brazelton TB, Yogman MW (1986). Discovery of an insecure-disorganized/disoriented attachment pattern. Affective development in infancy.

[12] Fonagy P, Steele M, Steele H, Moran GS (1991). The capacity for understanding mental states: the reflective self in parent and child and its significance for security of attachment. Infant Ment Health J.

[13] Steele H, Steele M, Fonagy P (1996). Associations among attachment classifications of mothers, fathers, and their infants. Child Dev.

[14] Luyten P, Campbell C, Allison E, Fonagy P (2020). The mentalizing approach to psychopathology: state of the art and future directions. Annu Rev Clin Psychol.

[15] Haynos AF, Fruzzetti AE (2011). Anorexia nervosa as a disorder of emotion dysregulation: evidence and treatment implications. Clin Psychol.

[16] Tasca GA, Balfour L (2014). Attachment and eating disorders: a review of current research. Int J Eat Dis.

[17] Zachrisson HD, Skårderud F (2010). Feelings of insecurity: review of attachment and eating disorders. Eur Eat Rev.

[18] Abbate-Daga G, Gramaglia C, Amianto F (2010). Attachment insecurity, personality, and body dissatisfaction in eating disorders. J Nerv Ment Dis.

[19] Troisi A, Di Lorenzo G, Alcini S (2006). Body dissatisfaction in women with eating disorders: relationship to early separation anxiety and insecure attachment. Psychosom Med.

[20] Szalai TD, Czeglédi E, Vargha A (2017). Parental attachment and body satisfaction in adolescents. Child Fam Stud.

[21] Illing V, Tasca GA, Balfour L (2010). Attachment insecurity predicts eating disorder symptoms and treatment outcomes in a clinical sample of women. J Nerv Ment Dis.

[22] Amianto F, Abbate-Daga G, Morando S (2011). Personality development characteristics of women with anorexia nervosa, their healthy siblings, and healthy controls: what prevents and what relates to psychopathology?. Psychiatry Res.

[23] Gale CJ, Cluett ER, Laver-Bradbury C (2013). A review of the father–child relationship in the development and maintenance of adolescent anorexia and bulimia nervosa. Issues Compr Pediatr Nurs.

[24] Cortés-García L, Takkouche B, Seoane G (2019). Mediators linking insecure attachment to eating symptoms: a systematic review and meta-analysis. PLoS ONE.

[25] Gezelius CME, Wahlund B, Carlsson L, Wiberg B (2016). Adolescent patients with eating disorders and their parents: a study of self-image and outcome at an intensive outpatient program. Eat Weight Disord.

[26] Benjamin LS (1974). Structural analysis of social behavior. Psychol Rev.

[27] Benjamin LS (1996). A clinician-friendly version of the interpersonal circumplex: structural analysis of social behavior (SASB). J Pers Assess.

[28] Onis MD, Onyango AW, Borghi E (2007). Development of a WHO growth reference for school-aged children and adolescents. Bull World Health.

[29] Shaffer D, Gould MS, Brasic J (1983). A children’s global assessment scale (CGAS). Arch Gen Psychiatry.

[30] Birgegård A, Björck C, Clinton D (2010). Quality assurance of specialized treatment of eating disorders using large-scale internet-based collection systems: methods, results and lessons learned from designing the stepwise database. Eur Eat Disord Rev.

[31] American Psychiatric Association (2000). A diagnostic and statistical manual of mental disorders, DSM-IV.

[32] Hazan C, Shaver P (1987). Romantic love conceptualized as an attachment process. J Pers Soc Psychol.

[33] Levy KN, Kelly KM, Obegi JH, Berant H (2009). Using interviews to assess adult attachment. Attachment theory and research in clinical work with adults.

[34] Håkanson A, Tengström A (1996). Attachment style questionnaire. Översättning till svenska samt inledande utprovning.

[35] Tengström A,Håkanson A (1997) Attachment style questionnaire. Manual till den svenska versionen, Vers 2. Preprint. Maria Ungdom—forskningsenheten, Stockholm, Sweden

[36] Lundh A, Kowalski J, Sundberg CJ (2010). Children’s global assessment scale (CGAS) in a naturalistic clinical setting: inter-rater reliability and comparison with expert ratings. Psychiatry Res.

[37] Bird HR, Canino G, Rubio-Stipec M (1987). Further measures of the psychometric properties of the children’s global assessment scale. Arch Gen Psychiatry.

[38] Schorre BEH, Vandvik IH (2004). Global assessment of psychosocial functioning in child and adolescent psychiatry. Eur Child Adolesc Psychiatry.

[39] McWilliams N (2011). Psychoanalytic diagnosis: understanding personality structure in the clinical process.

[40] Gezelius CME, Wiberg B (2000). Gyllene ögonblick: Om “alternerande medvetandetillstånd” i psykoterapi, tidig mor/barnrelation och kreativitet. Insikten.

[41] Cortés-García L, McLaren V, Vanwoerden S (2021). Attachment, mentalizing, and eating disorder symptoms in adolescent psychiatric inpatients and healthy controls: a test of a mediational model. Eat Weight Disord.

[42] Jewell T, Blessitt E, Stewart C (2016). Family therapy for child and adolescent eating disorders: a critical review. Fam Process.

[43] Erikson EH (1985). The life cycle completed. A review.

[44] Monteleone AM, Mereu A, Cascino G (2021). The validity of the fifth and the 10th body mass index percentile as weight cut-offs for anorexia nervosa in adolescence: no evidence from quantitative and network investigation of psychopathology. Eur Eat Dis Rev.

[45] Ringer F, Crittenden PM (2007). Eating disorders and attachment: the effects of hidden family processes on eating disorders. Eur Eat Dis Rev.

